# Antacids, Altered Mental Status, and Milk-Alkali Syndrome

**DOI:** 10.1155/2012/942452

**Published:** 2012-12-30

**Authors:** Simon C. Watson, Bonnie B. Dellinger, Katie Jennings, Lancer A. Scott

**Affiliations:** Division of Emergency Medicine, Medical University of South Carolina, 169 Ashley Avenue, MSC 300 Room 294, Charleston, SC 29425, USA

## Abstract

The frequency of milk-alkali syndrome decreased rapidly after the development of histamine-2 antagonists and proton pump inhibitors for the treatment of peptic ulcer disease; however, the availability and overconsumption of antacids and calcium supplements can still place patients at risk (D. P. Beall et al., 2006). Here we describe a patient who presented with altered mental status, hypercalcemia, metabolic alkalosis, and acute renal failure in the context of ingesting large amounts of antacids to control dyspepsia.

## 1. Introduction

Milk-alkali syndrome is caused by excessive ingestion of calcium and base, usually in the form of milk and antacids. It was first described in the 1920s with the advent of a new treatment for peptic ulcer disease, the Sippy regimen, that involved milk, bicarbonate, and calcium carbonate [1]. Now, because of the availability and abundance of over-the-counter antacid medications, several cases have been reported [2, 3]. This syndrome is classically characterized by hypercalcemia, metabolic alkalosis, and renal failure. Severity of this syndrome can range from mild gastrointestinal discomfort to death and requires the ED physician to have knowledge of the syndrome and to maintain a high level of suspicion in patients presenting with hypercalcemia. 

## 2. Case Report

A 68-year-old white male with a history of hypertension, gastroesophageal reflux disease, and congestive heart failure was found by his son lying on the floor of his home. The son activated the EMS system, and the paramedics found the patient with altered mentation, responsive to pain and mumbling. The patient was incontinent of feces and urine. The son last spoke to the patient two days earlier and reports that he was in his normal health. The patient's history included depression, benzodiazepine dependence, and anxiety disorder. The patient's daily medications included furosemide 40 mg, isosorbide dinitrate 30 mg, lisinopril 20 mg, and atorvastatin 10 mg. The patient was known to ingest large amounts of over-the-counter antacids and baking soda mixed with water for indigestion. His social history was significant for tobacco and alcohol. 

Upon arrival to the emergency department (ED) the patient's blood pressure was 218/111, the cardiac monitoring showed sinus tachycardia at 127/minute, a rectal temperature 98.6°F was measured, the room air SPO2 was 94%, and respirations were 16/minute. The patient was able to follow simple commands. He opened his eyes to voice and moved his extremities, including pulling his EKG wires. The patient had pale skin, lateral nystagmus, and signs of poor hygiene. The remainder of the physical exam was unremarkable except for abdominal pain, nausea, vomiting, or generalized pain. The patient was given labetalol 10 mg for hypertension and aggressive intravenous fluid hydration and IV folic acid, magnesium sulfate, multivitamin, and thiamine. 

Significant laboratory results included calcium of 18.7 mg/dL, bicarbonate 45 mmol/L, BUN 55 mg/dL, creatinine 4.5 mg/dL, chloride 83 mmol/L, and WBC 22.46 K/cumm. Arterial blood gas revealed a pH of 7.54, pCO_2_ of 58 mmHg, and pO_2_ of 60 mmHg. Parathyroid hormone was undetectable (normal range is 14–72). Liver function tests, coagulation studies, and lipase were normal. EKG showed sinus tachycardia and head CT showed no acute changes.

The patient was admitted to the intensive care unit with a BP of 184/116, pulse 88/min, and respirations of 16/min. A chest X-ray showed infiltrates, concerning for pneumonia. The patient was placed on a nitroglycerin drip to control his blood pressure, antibiotics for presumed infection, IV fluids and a bisphosphonate for hypercalcemia, and risperidol for signs of delirium. 

**Figure 1:**
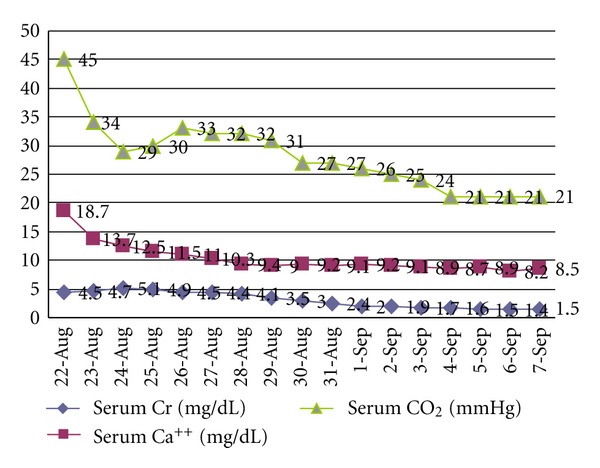
Trend of Patient's metabolic alkalosis, hypercalcemia, and acute renal failure throughout hospital admission (8/22–9/7).

The patient was admitted for a total of 16 days. Upon discharge the patient's mental status had returned to nearly normal, the patient was found to have an underlying dementia not previously diagnosed. The discharge blood values showed calcium 8.5 mg/dL, bicarbonate 21 mmol/L, BUN 19.0 mg/dL, creatinine 1.4 mg/dL, and WBC 10 K/cumm.  Figure 1 demonstrates the progressive resolution of the patient's metabolic alkalosis, hypercalcemia, and renal failure throughout this hospital course. The patient was placed on Protonix for reflux, given instruction to avoid antacid use and to follow up with the internal medicine clinic. 

## 3. Discussion 

A retrospective chart review of renal disease inpatients in 2005 identified 125 patients with hypercalcemia [4]. Milk-alkali syndrome accounted for 9% of these patients making it the third most common cause, behind malignancy and hyperparathyroidism. 

The incidence of milk-alkali syndrome had previously decreased with the use of Histamine-2 acid suppressing and proton pump inhibiting medications. However, recent medical literature indicates increases in patients presenting with this syndrome [3, 5–12]. This may be attributable to increased physician awareness in diagnosis and subsequent reporting, but also the ease of availability in many over-the-counter antacid preparations containing calcium carbonate. Like many cases described in the literature, the patient discussed here was using an antacid for dyspepsia. Other risk factors include female gender, hypertension, chronic kidney disease, use of diuretics, osteoporosis, and upper gastrointestinal disease [4]. 

The pathophysiology of milk-alkali syndrome is a complicated process leading to alterations in calcium homeostasis and hypercalcemia. Excessive intake is thought to be greater than 4 g of calcium carbonate daily [13]. Hypercalcemia leads to vasoconstriction of the afferent renal arterioles and thus a decrease in the glomerular filtration rate (GFR). A decrease in GFR subsequently leads to renal insufficiency which further exacerbates the ability of the kidney to excrete calcium and leads to increasingly higher serum calcium levels. Hypercalcemia also leads to decreased sodium reabsorption and an increased free water clearance, leading to volume depletion. The renal tubules then reabsorb bicarbonate in an effort to counteract this volume depletion, further contributing to the metabolic alkalosis found in milk-alkali syndrome [2, 4].

Because presentation of milk-alkali syndrome is widely variable, a careful history is important to making the correct diagnosis. A classically taught mnemonic used to recall effects of hypercalcemia lists bones (osteoporosis), stones (nephrolithiasis), groans (abdominal pain, nausea, vomiting), moans (bone pain), and psychic overtones (depression) as clinical symptoms. Other attributed signs and symptoms included, headache, vomiting, drowsiness, dehydration, weakness, elevated heart rate, disorientation, and altered mental status [6, 7, 9, 14].

A review of over-the-counter medications will often reveal the source of increased calcium intake. Laboratory values are also an important part of the diagnosis and include elevated calcium, a low or normal PTH level, and a metabolic alkalosis. 

Once the diagnosis is made, emergent treatment should include stopping the offending agent and administering aggressive IV fluids [2, 4]. In most cases, this alone will correct the patient's metabolic derangements; however, severe cases may require the addition of furosemide, bisphosphonates, steroids [2, 4], and calcitriol [4, 15]. Historically, furosemide has been used after volume status has been restored in order to promote calciuresis; however, more recent studies have been unable to support the effectiveness of furosemide as first line therapy for hypercalcemia [15]. Bisphosphonates, such as pamidronate and zoledronate, may take 24–48 hours to take effect and thus are not to be used as first-line agents in the emergency department. A common complication of bisphosphonate use is hypocalcemia so calcium levels should be monitored closely when employing this therapy. Calcitriol may decrease calcium levels in as little as 2 hours when used IM or SQ, making it potentially well suited in the acute care setting.

## 4. Conclusions 

Milk-alkali syndrome can be a difficult diagnosis that requires a high index of suspicion in order to quickly identify the disorder and initiate appropriate therapy. As a common cause of hypercalcemia, it is important for clinicians to keep the syndrome on their list of differential diagnoses. Failure to recognize the syndrome or appreciate its dangers can lead to prolonged hospital stays, permanent kidney damage, neurologic damage, and death. Since patient presentation alone can be highly variable and vague, a careful history and knowledge of the syndrome are key factors in caring for patients with milk-alkali syndrome.
